# Baseline Magnetic Resonance Imaging of the Optic Nerve Provides Limited Predictive Information on Short-Term Recovery after Acute Optic Neuritis

**DOI:** 10.1371/journal.pone.0113961

**Published:** 2015-01-30

**Authors:** Sebastian Berg, Iris Kaschka, Kathrin S. Utz, Konstantin Huhn, Alexandra Lämmer, Robert Lämmer, Anne Waschbisch, Stephan Kloska, De-Hyung Lee, Arnd Doerfler, Ralf A. Linker

**Affiliations:** 1 Department of Neurology, University Hospital Erlangen, University of Erlangen-Nuremberg, Erlangen, Germany; 2 Department of Neuroradiology, University Hospital Erlangen, University of Erlangen-Nuremberg, Erlangen, Germany; 3 Department of Ophthalmology, University Hospital Erlangen, University of Erlangen-Nuremberg, Erlangen, Germany; Medical University of Innsbruck, AUSTRIA

## Abstract

**Background:**

In acute optic neuritis, magnetic resonance imaging (MRI) may help to confirm the diagnosis as well as to exclude alternative diagnoses. Yet, little is known on the value of optic nerve imaging for predicting clinical symptoms or therapeutic outcome.

**Purpose:**

To evaluate the benefit of optic nerve MRI for predicting response to appropriate therapy and recovery of visual acuity.

**Methods:**

Clinical data as well as visual evoked potentials (VEP) and MRI results of 104 patients, who were treated at the Department of Neurology with clinically definite optic neuritis between December 2010 and September 2012 were retrospectively reviewed including a follow up within 14 days.

**Results:**

Both length of the Gd enhancing lesion (r = -0.38; p = 0.001) and the T2 lesion (r = -0.25; p = 0.03) of the optic nerve in acute optic neuritis showed a medium correlation with visual acuity after treatment. Although visual acuity pre-treatment was little but nonsignificantly lower if Gd enhancement of the optic nerve was detected via orbital MRI, improvement of visual acuity after adequate therapy was significantly better (0.40 vs. 0.24; p = 0.04). Intraorbitally located Gd enhancing lesions were associated with worse visual improvement compared to canalicular, intracranial and chiasmal lesions (0.35 vs. 0.54; p = 0.02).

**Conclusion:**

Orbital MRI is a broadly available, valuable tool for predicting the improvement of visual function. While the accurate individual prediction of long-term outcomes after appropriate therapy still remains difficult, lesion length of Gd enhancement and T2 lesion contribute to its prediction and a better short-term visual outcome may be associated with detection and localization of Gd enhancement along the optic nerve.

## Introduction

Optic neuritis (ON) is defined as an inflammatory condition of the optic nerve resulting in acute visual impairment. Leading symptoms include reduced visual acuity, visual field defects, periocular pain—especially during eye movement-, reduced contrast sensitivity and dysfunction of color vision [1–3]. ON is typically diagnosed in young adults aging 20–45 years, with women being three times more often affected than men [4, 5]. The etiology of acute demyelinating ON is commonly idiopathic or autoimmune. In addition, toxic, nutritional, metabolic, vascular, hereditary, infectious, infiltrative, or compressive conditions may lead to similar symptomatology [1, 2, 4, 6]. In 15–25 percent of patients suffering from multiple sclerosis (MS), ON is the first clinical event and 50–60 percent develop ON at some point during disease course [7–10]. Cranial magnetic resonance imaging (cMRI) including optic nerve imaging may help to rule out alternative diagnosis and determine the risk for developing MS [11–15]. Optic nerve lesions may be detected by orbital MRI including fat-saturated fast spin-echo (FSE) imaging and fat-saturated T1-weighted imaging following gadolinium with strong sensitivity [16–20]. Although long-term visual outcome of ON is generally favorable, about one in three patients remains visually impaired [11, 14]. A few studies suggest that poor visual outcome is associated with more extensive or intracanalicular lesions in orbital MRI, lower VEP amplitude or worse initial visual impairment [17, 21, 22]. Modern techniques such as optical coherence tomography (OCT) or multifocal visual evoked potential (mfVEP) are beliefed to reflect optic nerve axonal degeneration and demyelination and might predict visual outcome after ON [23–26]. However, while retinal nerve fibre layer (RNFL) thickness measured by OCT can be confounded by inflammatory edema, VEP may be completely extinguished due to acute demyelination and associated conduction block. Hence, other techniques are needed for prediction of visual outcome after ON. This study aims to evaluate the benefit of optic nerve MRI for predicting response to appropriate therapy and normalizing visual acuity.

## Methods

### Patients and neurological exam

From December 2010 to September 2012, a total of 104 patients with ON were treated at the Department of Neurology at the University Hospital Erlangen, Germany. Optic neuritis was clinically diagnosed and defined by a typical history and typical clinical features as acute visual impairment, ranging from only light perception to 1.0, pain in or around the affected eye on eye movements, impaired color vision, afferent pupillary defect and central or paracentral scotoma. Diagnosis was confirmed by independent neurological and ophthalmological examination. Patients were investigated according to the hospital routine standard investigation protocol for optic neuritis. This protocol was performed within 24 hours of admission and contains detailed ophthalmological examination including visual acuity, refraction, pupillary function, ocular motility, visual field testing, slit lamp, tonometry and fundus examination after pupil dilation to exclude macular, retinal or optic disc disorders, standard blood analysis including differential blood count, creatinine, liver enzymes, coagulation parameters, CRP, ACE, serology for borrelia and lues, anti-aquaporin-4 antibodies, ANA screen, vitamin B12, orbital MRI and visual evoked potentials. Patients with history for any condition that could explain symptoms similar to optic neuritis underwent further investigations to rule out differential diagnoses and were excluded from the analysis. Patients with optic neuropathy secondary to tumor, vascular disease, diabetes, infectious agents or toxins were excluded, as well as patients with papillitis, perineuritis, sarcoidosis, Morbus Behçet, chronic relapsing inflammatory optic neuropathy, neuromyelitis optica or previous episodes of ipsilateral optic neuritis. Visual acuity was measured using the standard Snellen chart and was recorded as decimal equivalent (e.g. 20/20 = 1.0). Patients neither diagnosed with MS nor clinically isolated syndrome who showed no MRI lesion outside the optic nerve were diagnosed as having isolated optic neuritis.

After exclusion of contraindications and after MRI, patients received a steroid pulse therapy with 1000mg or 2000mg methylprednisolone per day over a period of three to five days. Patients with remaining severe visual impairment (visual acuity < 0.5) despite previous steroid pulse therapy within the last 6 weeks were treated with plasma exchange every other day for at least 5 cycles according to established protocols [27, 28]. A final neuro-ophthalmologic examination was performed within 14 days after adequate therapy consisting of neurological status, testing of visual function and visual evoked potentials.

### MRI technique

Cranial and orbital MR imaging was performed within the first 24 hours after admission to the hospital. MRI of the brain was performed on a 1.5T or 3T unit (MAGNETOM Aera or MAGNETOM Trio TIM; Siemens AG, Erlangen, Germany) with an 8 channel head coil. The MR imaging parameters are summarized in table 1. The contrast enhanced T1-weighted sequences were acquired with a delay of at least 5 minutes after the intravenous injection of the contrast agent (Gadovist, 0.1mmol/kg). The images were evaluated independently by two neuroradiologists who were unaware of the clinical findings (IK, SK). Cranial MRI was evaluated in consideration of signal abnormalities suggestive of lesions typical for demyelination in the brain [17]. Location and dissemination in space were evaluated according to the Swanton-criteria (infratentorial, juxtacortical, periventricular, spinal) [29]. On orbital MRI, location (intraorbital, canalicular, intracranial, chiasmal) and size of edema as well as contrast enhancement of the optic nerve were analyzed [17]. Length of contrast enhancement and edema were determined on thin layered coronar fat-saturated orbital sequences as the number of consecutive slices involved multiplied by 3mm.

**Table 1:** MR imaging parameters.

	**Cerebrum**					**orbita**	
**sequences**	T2 3D FLAIR	T1 spin-echo	T2 turbo spin-echo	DWI	T1 spin-echo post C	fat-saturated T2 turbo spin-echo	fat-saturated T1 spin-echo post C
TR	5000	407	3070	6800	407	4380	500
TE	333	8.8	107	89	8.8	98	9.8
TI	1800						
Orientation	sagittal	transversal	Transversal	transversal	transversal and coronar	coronar	transversal and coronar
slice thickness [mm]	0.6	5	5	5	5	3	3
reconstructed slice thickness [mm]	3 (sagittal)5 (transversal and coronar)						

### Statistics

Data were retrospectively reviewed focusing on relation of visual function with MRI-results. Statistical differences were calculated via Mann Whitney rank sum test and defined by p<0.05 (GraphPad Prism version 5.03). Non parametric Spearman correlations were calculated using SPSS version 20.0 (IBM Software, USA).

### Regulatory issues

All authors involved in this study are MD at the Departments of Neurology, Ophthalmology or Neuroradiology of the University Hospital Erlangen and thus were involved in the treatment of patients and had access to identifying information as well as patient medical records. Data were acquired in the context of routine patient treatment at the Neuroimmunology Clinics of the Department of Neurology. For this routine data basing, any patient does give written informed consent upon first contact with the Department which is in complete accordance with the National data protection law and the local Ethics Committee.

For the purpose of this study, a retrospective search was performed on this database by the Department of Informatics of the University Hospital in accordance with the Ethics Committee of the Medical Faculty of the University of Erlangen-Nuremberg. Beforehand, the Ethics Committee did evaluate the entire study design, methods and data access protocol and granted a specific waiver to conduct this study, and also waived the need to obtain written informed consent from the patients again. Patient data were anonymized by the first author of the study prior to analysis. All patients provided their written informed consent for their MRI scan images to be used as representative illustrations in this research article.

## Results

### Baseline data and cranial MRI results

A total of 104 patients with clinically definite optic neuritis were included, 70 (67.3%) of which were female and 34 (32.7%) were male, with a median age of 37 years. The mean visual acuity of all patients before treatment was 0.47 ranging from only light perception to 1.0. Follow up could be completed in 94 patients, mean visual acuity 14 days after treatment was 0.83. After careful analysis of brain and spinal MRI and patient history 48 (46.2%) patients were diagnosed with MS according to the 2010 revision of the McDonald criteria, 27 (26.0%) were diagnosed with clinically isolated syndrome and 29 (27.9%) were diagnosed with isolated ON, defined as a clinically definite ON with absence of inflammatory brain lesions. 56 (53.8%) patients showed dissemination in space of inflammatory lesions in T2-Flair-weighted MRI-sequences according to the 2010 revised McDonald multiple sclerosis diagnostic criteria. 48 (46.2%) patients did not show dissemination in space (data are summarized in Table 2).

**Table 2:** Characteristics of the study cohort.

Female	70	67.3%
Male	34	32.7%
Median age (years)	37	range 20…83
Ethnicity: Caucasian	104	100%
Isolated ON	29	27.9%
Clinically isolated syndrome	27	26.0%
Definite multiple sclerosis (2010 revison of the McDonald criteria)	48	46.2%
Swanton criteria positive	56	53.8%
Swanton criteria negative	48	46.2%
T2 lesion of optic nerve	83	79.8%
Gd enhancement of optic nerve	77	74.0%
Patients receiving plasmapheresis	12	11.5%
Mean visual acuity pre treatment	0.47	± 0.03 (SEM)
Mean visual acuity post treatment	0.83	± 0.04 (SEM)
Mean VEP P100 latency of affected eye pre treatment (n = 99)	113.2 ms	± 4.1 (SEM)
Mean VEP P100 amplitude of affected eye pre treatment (n = 97)	4.4 µV	0.3 µV (SEM)
Mean P100 latency of affected eye post treatment (n = 36)	124.8 ms	± 2.1 (SEM)
Mean VEP P100 amplitude of affected eye pre treatment (n = 97)	6.4 µV	0.6 µV (SEM)
Median time from onset of ON to MRI (days)	5	range 0…87

### Results of optic nerve imaging

**Figure 1:**
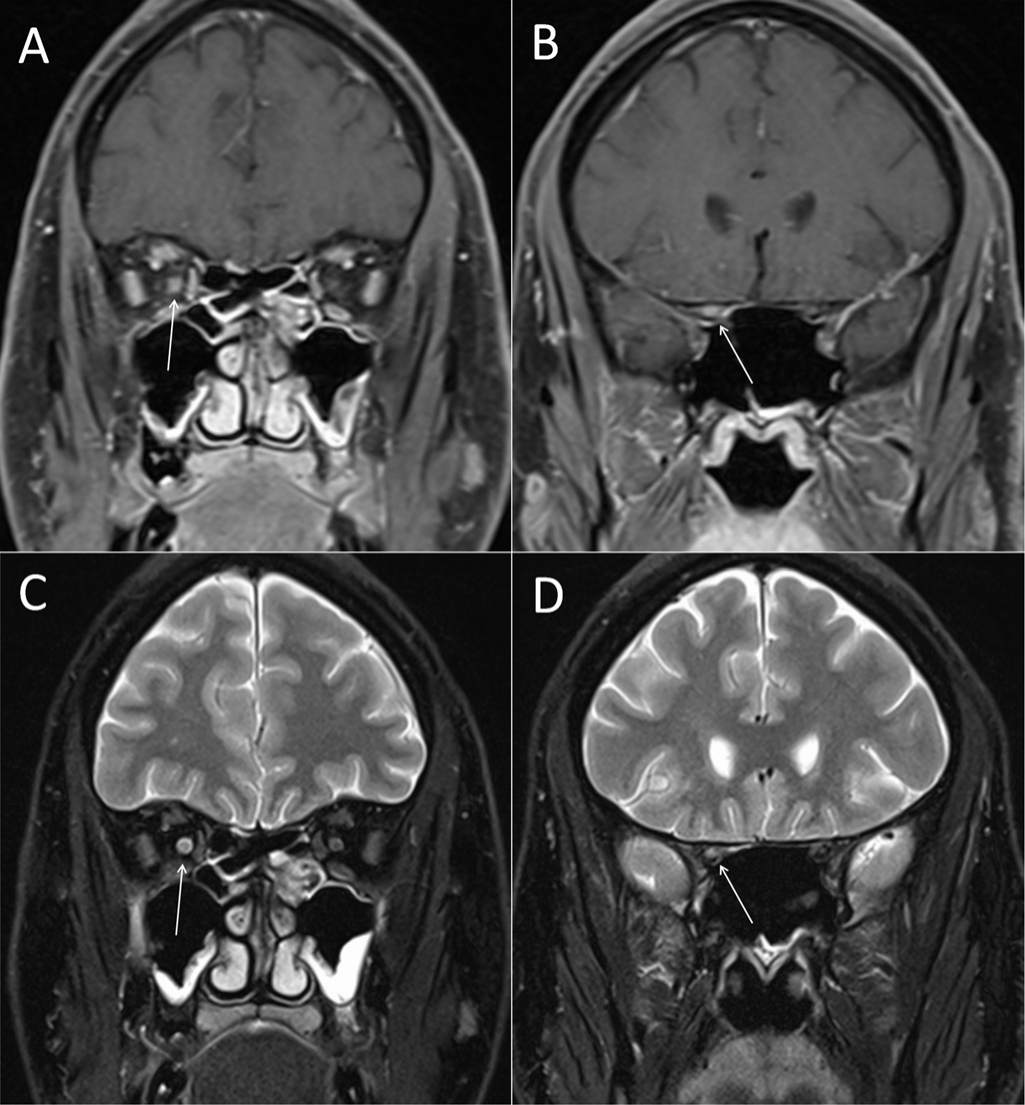
Gd enhancement and T2 lesion of the right optic nerve. Optic nerve MR imaging of a representative patient with ON. (A) Coronar fat-saturated T1-weighted MRI sequences of the intraorbital and (B) canalicular part of the optic nerve after application of 0.1mmol/kg gadolinium. The arrows highlight the Gd enhanced right optic nerve. (C) Signalalteration of the intraorbital and (D) canalicular part of the optic nerve in coronar fat-saturated T2 turbo spin-echo MRI sequences. The arrows highlight the hypertense T2 lesion of the right optic nerve.

We examined the patients for edema or Gd enhancement of the optic nerve in thin layered orbital MRI. Of all patients with clinically diagnosed ON, 83 (79.8%) showed signal alterations of the optic nerve in T2-FSE-weighted sequences of the orbital MRI consistent with edema of the optic nerve while 77 (74.0%) patients showed Gd enhancement of the optic nerve (see Fig. 1A–1D). The presence of both, T2 lesion and Gd enhancement of the optic nerve was found in 72 (69.2%) patients (Table 2).

The finding of inflammatory lesions in T2-Flair-weighted MRI in at least two of the Swanton locations showed no significant relation with pathological findings of optic nerve imaging in orbital MRI. Similarly, there was no significant difference in VEP latencies or amplitudes between patients with or without T2 lesion or Gd enhancement of the optic nerve (not shown).

Upon analysis by visual categories, 95.0% of patients with severe visual impairment (<0.1) were positive for T2 lesion of the optic nerve, yet there was no significant difference between patients with or without T2 lesion or Gd enhancement for any category of visual impairment (Table 3).

**Table 3:** Visual categories (acc. ONTT).

**Visual acuity before treatment**	**total**	**T2 lesion (n = 83)**	**Gd+ (n = 77)**
0…<0.1	20	19 (95.0%)	17 (85.0%)
0.1…<0.2	10	7 (70.0%)	7 (70.0%)
0.2…<0.5	19	15 (79.0%)	14 (73.7%)
0.5…<0.8	32	26 (81.0%)	24 (75.0%)
0.8…≤1.0	23	16 (70.0%)	15 (65. 2%)

Plasma exchange therapy was performed in 12 patients, of which 10 showed T2 lesion of the optic nerve and 7 presented Gd enhancement. This group did not significantly differ from patients only receiving steroid pulse therapy regarding positive Swanton criteria or the diagnoses of MS, clinically isolated syndrome or isolated optic neuritis (Table 4).

**Table 4:** Characteristics of patients receiving plasma exchange therapy.

	**Total (n = 12)**	**T2 lesion (n = 10)**	**Gd+ (n = 7)**
Isolated ON	2 (16.7%)	1 (10.0%)	1 (14.3%)
Clinically isolated syndrome	3 (25.0%)	2 (20.0%)	2 (28.6%)
Definite multiple sclerosis (2010 revision of the McDonald criteria)	7 (58.3%)	7 (70.0%)	4 (57.1%)
Swanton positive	6 (50.0%)	6 (60.0%)	5 (71.4%)
Swanton negative	6 (50.0%)	4 (40.0%)	2 (28.6%)
Mean visual acuity pre treatment	0.22 ± 0.10 SEM	0.16 ± 0.08 SEM	0.11 ± 0.04 SEM
Mean visual acuity post treatment	0.53 ± 0.07 SEM	0.24 ± 0.07 SEM	0.22 ± 0.06 SEM
Mean VEP P100 latency of affected eye pre treatment	104.9 ms ± 15.9 SEM	103.2 ms ± 17.4 SEM	131.6 ms ± 2.9 SEM
Mean VEP P100 amplitude of affected eye pre treatment	2.3 µV ± 0.6 SEM	2.1 µV ± 0.7 SEM	2.4 µV ± 0.8 SEM
Mean VEP P100 latency of affected eye post treatment	123.0 ms ± 4.5 SEM	125.0 ms ± 4.8 SEM	124.5 ms ± 7.3 SEM
Mean VEP P100 amplitude of affected eye post treatment	4.8 µV ± 1.2 SEM	5.3 µV ± 1.4 SEM	6.1 µV ± 1.8 SEM

In all patients, the median time from onset of optic neuritis to MRI procedure and VEP examination was 5 days. When comparing patients with or without T2 lesion of the optic nerve, the median time of onset to MRI/VEP was not significantly different (6 vs. 4 days, p = 0.27). Yet, the median time of onset of optic neuritis to MRI/VEP procedure tended to be shorter in patients showing Gd enhancement of the optic nerve as compared to patients without Gd enhancement but was not statistically significant. (5 vs. 8 days, p = 0.06)

### Predictive value of initial orbital MRI on short-term follow-up improvement

**Figure 2:**
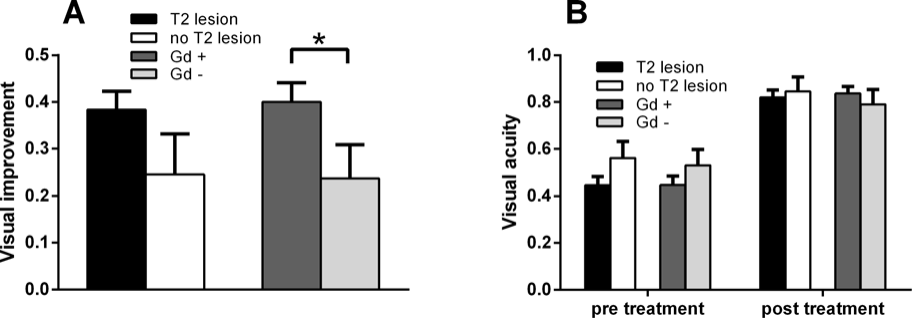
Patients with Gd enhancement of the optic nerve show significant higher visual improvement. (A) Mean improvement of visual acuity of patients with or without either T2 lesion or Gd enhancement of the optic nerve in orbital MRI. Error bars indicate standard error of the mean. * indicates significant difference with p = 0.04 as determined via Mann-Whitney rank sum test. Improvement of visual acuity was defined as difference between visual acuity before and after adequate treatment. (B) Mean visual acuity of patients with or without T2 lesion or Gd enhancement of the optic nerve in orbital MRI before and after adequate therapy. Error bars indicate standard error of the mean. No significant differences were found between the single groups.

Within 14 days after adequate therapy with methylprednisolone and/or plasma exchange therapy patients underwent a follow-up neuro-ophthalmic examination. After treatment the mean visual acuity in all patients improved from a 0.47 to 0.83 (see also Table 2). We sought for a relation between radiological characteristics of optic nerve imaging and clinical outcomes and VEP outcomes. The mean improvement of visual acuity—defined as the difference of visual acuity before and after adequate therapy—was significantly better if Gd enhancement of the optic nerve was detected on orbital MRI (0.40 vs. 0.24; p = 0.04). In addition, there was a tendency towards a better visual improvement when orbital MRI showed T2 lesion of the optic nerve which was not statistically significant (0.38 vs. 0.25; Fig. 2A).

Upon group analysis mean visual acuity before and after adequate therapy did not significantly differ between patients who showed T2 lesion respectively contrast enhancement of the optic nerve and those who did not (Fig. 2B).

We also assessed the location and lesion length of T2 lesion as well as Gd enhancement along the optic nerve. Lesion locations were grouped into intraorbital, canalicular, intracranial and chiasmal lesions. There was a significant difference between the mean visual improvement of patients with intraorbital Gd enhancing lesions and patients with Gd enhancing lesions elsewhere (0.35 vs. 0.54; p = 0.02; Table 5). No other significant differences in lesion location of T2 lesion or Gd enhancement and visual acuity have been observed.

**Table 5:** Clinical Parameters in relation to MRI results.

	**T2 lesion of optic nerve**					**Gd enhancement of optic nerve**				
	n	Mean visual acuity pre treatment	Mean visual acuity post treatment	Mean visual improvement	Median time onset of ON to MRI	n	Mean visual acuity pre treatment	Mean visual acuity post treatment	Mean visual improvement	Median time onset of ON to MRI
Total	83	0.45 ± 0.04 SEM	0.82 ± 0.03 SEM	0.38 ± 0.03 SEM	6 days	77	0.45 ± 0.04 SEM	0.84 ± 0.03 SEM	0.40* ± 0.04 SEM	5 days
lesion length										
3–9mm	32	0.45	0.80	0.36	7 days	44	0.46	0.91**	0.48*	4.5 days
12–15mm	26	0.46	0.87	0.41	6 days	17	0.38	0.77	0.37	5 days
18–24mm	15	0.47	0.85	0.44	8 days	11	0.57	0.82	0.25*	9 days
27–36mm	10	0.37	0.48***	0.07**	9 days	5	0.30	0.52*	0.21	5 days
lesion localisation										
intraorbital	69	0.45	0.82	0.38	7 days	55	0.46	0.80	0.35*	5 days
canalicular	31	0.43	0.80	0.38	5.5 days	34	0.47	0.85	0.39	6 days
intracranial	17	0.41	0.77	0.33	6 days	18	0.39	0.81	0.40	6 days
Chiasmal	6	0.60	0.78	0.18	7 days	3	0.57	0.90	0.33	7 days

*=p<0,05;

**=p<0,01;

***=p<0,001

p value was determined via Mann-Whitney rank sum test.

Short Gd enhancing lesions were associated with a significantly better visual acuity after treatment and very long Gd enhancing lesions as well as T2 lesions were associated with a significant worse visual acuity after treatment (Table 5). A non parametric correlation was calculated and showed a medium negative correlation between visual acuity after treatment and lesion length of Gd enhancing lesion (r = -0.38; p = 0.001) respectively lesion length of T2 lesion (r = -0.25; p = 0.03; Table 6). This means, the longer the T2 or Gd enhancing lesion on MRI, the poorer the visual acuity after treatment.

**Table 6:** Correlation between visual acuity after treatment and demographic, clinical, electrophysical and MRI parameters.

	**Length of T2 lesion**	**Length of Gd enhancing lesion**	**sex**	**age**	**time from onset to MRI**	**VEP latency**	**VEP amplitude**
r	-0,25	-0,3791	-0,05	-0,26	-0,24	0,08	0,26
p	0,03	0,0012	0,63	0,01	0,03	0,43	0,01
n	77	70	94	94	85	90	88

From all other demographic, clinical and VEP parameters, age (r = -0.26; p = 0.01), time from onset to MRI (r = -0.24; p = 0.03) and VEP amplitude (r = 0.26; p = 0.01) showed significant but low correlation with visual acuity after treatment (Table 6).

Visual acuity before treatment correlated with no other demographic, clinical and VEP parameters but with VEP amplitude (r = 0.54; p = <0.0001) and visual acuity after treatment (r = 0.27; p = 0.008).

## Discussion

In this study, we demonstrate that orbital MRI is a reliable tool to confirm the diagnosis of an optic neuritis and may help to predict visual outcome.

Most importantly, we demonstrate that the length of the Gd enhancing lesion as well as the T2 lesion correlates with the visual outcome after adequate treatment of ON. Furthermore, we show that detection and localization of Gd enhancement is associated with the visual outcome.

Gd enhancement as a sign for brain barrier breakdown indicates acute inflammation of the optic nerve [30–32]. It has been previously described in 94.4% of optic neuritis patients [18]. More recent studies suggested a lower sensitivity more consistent with our results (66.7% [33]; 85% [17]; 75% [34]; 74% in our study).

Signal alterations in fat-saturated T2-FSE weighted orbital imaging as a sign of edema of the optic nerve due to inflammation was found at a high percentage of patients presenting typical clinical symptoms of optic neuritis. In previous studies with smaller cohorts of 21 to 33 patients, the sensitivity of fat-saturated T2-FSE MRI to detect optic neuritis was 95.2% to 100% [16, 17]. Our results are consistent with a more recent study that showed edema of the optic nerve in 83.3% of optic neuritis patients [33]. A lower sensitivity of fat-saturated T2-FSE MRI may be due to a larger and therefore potentially more heterogeneous study cohort.

In 5 of the 104 cases in our study pathologic contrast enhancement of the optic nerve was seen in the absence of signal changes in the T2 weighted images. It can be hypothesized that these subjects had inflammation of the optic nerve in a subacute stage with resolved acute edema but residual contrast enhancement.

An initial MR based study related optic nerve imaging to visual recovery over time but did not focus on the relation of initial MR findings to clinical short-term improvement. Furthermore, there were only six cases without Gd enhancement of the optic nerve to compare with contrast enhancement thus possibly lowering the power of that study to detect significant differences [18]. Another study from the same group demonstrated an association between length of the contrast enhancing lesion and visual recovery [17]. Other analyses examining the relationship between edema of the optic nerve in fat-saturated T2-FSE weighted MRI and visual impairment demonstrated that poor recovery from optic neuritis was associated with more extensive lesions or intracranial lesions [21, 22]. Our study corrobates previous studies and reveals a negative correlation between visual outcome and lesion length of both Gd enhancing lesions as well as T2 lesion in a large cohort. We also show that Gd enhancement of the affected optic nerve in patients with ON is associated with a better short-term improvement of visual acuity after adequate therapy. This may in part be explained by a slightly lower initial visual acuity in the Gd enhancing group. Yet, pre-treatment visual acuity was not significantly different between both groups thus rendering this explanation rather unlikely. Gd enhancement of the optic nerve is detected in patients with a more acute onset of disease and a higher potential to prompt improvement of visual impairment.

In our study, visual acuity pre-treatment did not correlate with any clinical or MRI variable but with VEP amplitude, which has been shown multiple times in previous studies [17, 35–37].

Modern non-conventional MR imaging techniques such as diffusion tensor imaging (DTI) and magnetisation transfer ratio (MTR) were shown to be sensitive to optic nerve damage in patients with previous episodes of ON and DTI indices may correlate with impaired visual acuity and retinal nerve fiber layer thinning [38–40]. Studies examining serial DTI in the setting of acute ON showed that axial diffusivity (AD) at baseline may correlate with visual contrast sensitivity but not visual acuity at 1 month and 3 month follow up [41]. Another study from the same group revealed a moderate correlation between AD at baseline and visual contrast sensitivity as well as visual acuity at 6 month follow up. Furthermore a lower baseline AD seemed to be associated with incomplete 6-month visual recovery [42]. A more recent study could not reproduce correlations between baseline AD and visual outcome but showed that persistent AD reduction at 3 months after acute ON predicts poorer visual outcomes at 6 and 12 month follow up [43]. Other studies examining serial MTR imaging following acute ON demonstrated either an acute decrease of optic nerve MTR with no relationship to visual acuity or visual field mean deviation [44] or an delayed decrease of optic nerve MTR at 3 month with moderate association with poorer visual acuity at 6 months and 12 months [45]. Hickman et al. demonstrated an increased mean cross-sectional area of the intraorbital optic nerve at onset through 3 months which correlated with length of the contrast enhancing lesion, although optic nerve volume was not associated with visual outcome [46]. Although these studies show promising but inconsistent results for predicting visual outcome after acute ON, the implementation in standard clinical protocols is not yet feasible. Diffusion tensor imaging is time consuming and requires preparing and positioning of the patient along with an imaging session dedicated only to optic nerve imaging as well as expert rating of images whereas MTR imaging is quickly performed but fails to predict outcome at baseline. For both techniques 3 Tesla MRI scanner are required that are not available in most hospitals at the moment.

Our study provides only data from a short-term follow up 14 days after treatment, although definite visual outcome should better be evaluated at least 3 months after optic neuritis since some patients may need several weeks to recover. While these data are available from a subgroup of our patients, there is a significant drop out rate, especially of those patients with a better outcome, resulting in a much smaller study group and a significant bias in the retrospectively obtained results. We would thus opt to restrict our study to short term outcomes.

Given that orbital MRI is broadly available and already routinely used to confirm diagnosis of ON in standard clinical protocol it has also significant value as predictor of visual outcome, whereas other techniques like DTI, MTR, VEP and OCT are either not yet feasible in standard clinical protocol or need to be performed with some time lag to reliably predict visual outcome. While our study has some limitations due to its retrospective nature and its short-term follow up, the results from a large cohort suggest the implication of modern orbital MRI sequences in standard clinical routine to obtain relevant information for diagnosis, therapy and prognosis of ON. Prospective studies with longer follow up will be needed to further confirm these findings.
